# An Online International Collaborative Learning Program During the COVID-19 Pandemic for Nursing Students: Mixed Methods Study

**DOI:** 10.2196/34171

**Published:** 2022-01-24

**Authors:** Dukyoo Jung, Jennie C De Gagne, Eunju Choi, Kyuri Lee

**Affiliations:** 1 College of Nursing Ewha Womans University Seoul Republic of Korea; 2 School of Nursing Duke University Durham, NC United States; 3 College of Nursing University of Iowa Iowa City, IA United States

**Keywords:** COVID-19, distance education, global competencies, nursing students, program evaluation, synchronous virtual classroom, video conferencing

## Abstract

**Background:**

Given the limitations imposed by the COVID-19 pandemic, a better understanding of how nursing programs around the globe have implemented distance education methods and related initiatives to provide international collaborative learning opportunities as well as complementary aspects of practical education would be constructive for nursing students. It is expected that international collaboratives through web-based communication will continue to be increasingly utilized after the pandemic; therefore, it is time to discuss the effects and direction of these developments.

**Objective:**

We aimed to examine the impact of an online international collaborative learning program on prelicensure nursing students’ international and global competencies in South Korea.

**Methods:**

We conducted a mixed methods study (web-based surveys and focus group interviews). A total of 15 students participated in the study. The surveys were used to examine changes in participants’ global leadership competencies, and the focus group interviews were used to evaluate the program’s effectiveness and to identify opportunities for improvement. The online international collaborative program consisted of 7 synchronous web-based classroom sessions. Each session ran for 60 to 90 minutes. Faculty experts and nurses working in the United States discussed various topics with students, such as nursing education in the United States and evidence-based teaching and learning. The students gave presentations on the South Korean nursing education system. Data were analyzed with descriptive statistics, the Mann-Whitney U test, and content analysis methods.

**Results:**

Participants reported improvement in their global leadership competencies. Four main categories emerged from analysis of the focus interviews: (1) realistic applicability, (2) clarification, (3) expansion of perspectives, and (4) initiative.

**Conclusions:**

The online international collaborative learning program had a positive impact on the development of students’ international competencies. The findings support the further development of international exchange programs through web-based meetings in the postpandemic era.

## Introduction

Since the first COVID-19 infection was diagnosed in South Korea, rapid changes designed to contain the spread of the virus have affected all aspects of society [1]. The implementation of social distancing, isolation, and quarantines have forced people to work, learn, teach, and pursue activities of daily life while avoiding direct face-to-face contact as much as possible [2,3]. The shift away from direct personal engagement has been particularly evident in education as learning methods have expanded to include approaches conducted online and remotely [2,3]. Distance education has transitioned from being considered a learning method with future potential to becoming a common and accepted response to a global need [4-6]. Universities are embracing this trend toward distance learning by striving to strengthen online teaching expertise and learning capabilities and by progressing projects to develop a web-based education infrastructure necessary for the contactless era [7].

University international exchange programs have been especially affected by the COVID-19 pandemic [7]. Whereas international exchanges formerly consisted primarily of on-site in-person interactions, the prolonged COVID-19 outbreak has restricted movement between countries, making it difficult to develop global competencies through in-person visits and necessitating alternative means of conducting exchanges, such as videoconferences [7]. Although exchange programs have faced challenges to participation due to cost issues [8] and time constraints [9,10], strengthening the global competencies of nursing students remains of vital importance. The American Association of Colleges of Nursing [11] maintains that a nursing curriculum that reflects cultural competency factors contributes to an understanding of patient values and preferences as well as respect for and positive attention to patient needs.

Attempts to promote and enhance global competencies via international exchange have taken various forms. The Korean Accreditation Board of Nursing Education [12] has added awareness of domestic and international health policy changes to learning outcomes that lead to Nursing Education Accreditation, an addition reflected in curriculum revisions. The new recommendations have led South Korean nursing colleges to incorporate international collaborative learning activities into curriculum, including special lectures about or participation in diverse program activities [13], visits to low-income countries to observe health education practices [9], or training at advanced education institutions [10].

Despite the importance of international exchange programs in developing nursing students’ global competencies, there has been insufficient research to evaluate program content and learning demands. In a study [8] that compared the need for international exchange in nursing students from the United States, Vietnam, and South Korea, those from South Korea showed the high willingness to learn about international employment trends and the effects of the movement of health care personnel. Furthermore, nursing educators in Korea who were surveyed stressed that nursing students needed preparation to respond to the globalization of health and health care, and that global health competencies should be integrated into the undergraduate nursing curriculum [14]. Previous research suggests that international exchange programs should (1) allow students to obtain answers to their questions about international nursing activities through direct communication with nurses who are currently working in the international arena, and (2) set specific goals for nursing students who desire to become global nurses.

Global nursing competencies are reinforced as students from different nursing education systems exchange information and ideas, compare and weigh differences and similarities in nursing curricula, broaden their perspectives, and develop more mature critical thinking abilities [15]. Given the limitations imposed by the COVID-19 pandemic, a better understanding of how nursing education programs around the globe have implemented distance education methods and related initiatives to provide international collaboratives as well as complementary aspects of practical education would be constructive. Although the effects of online international exchange programs have not yet been reported, it is expected that international collaboratives through web-based communication will continue to be increasingly utilized after the COVID-19 pandemic; therefore, it is time to discuss the effects and direction of these developments. We aimed to analyze the effects of changes in educational delivery methods due to the COVID-19 pandemic on prelicensure nursing students participating in online international collaborative learning programs.

## Methods

### Study Aim and Design

A mixed method design was used in this study. We used quantitative research methods to evaluate the program quality of online international collaborative learning programs and the global leadership competencies of nursing student participants; we used qualitative research methods (focus group interviews) to explore the impact of participating in the program.

### Participants

Undergraduate students applied to and participated in the online international collaborative learning program led by the Office of International Affairs at Ewha Womans University. Students who completed the program with at least 70% attendance were eligible to be included in the study. All 16 students were eligible to participate and were offered the opportunity to participate in the study. Of them, 15 agreed to participate in the study.

### Instrument

#### Global Leadership Competencies

Global leadership is a competency that positively influences the thoughts, attitudes, and behaviors of stakeholders beyond the national, cultural, and linguistic differences based on open-mindedness and diversity for organizational growth [16,17]. To measure the global leadership competencies of undergraduate nursing students, we used a previously developed tool [16], based on 5 global leadership competencies [17], that consists of 18 questions comprising 5 subthemes: global mind (3 questions), open attitude toward diversity (4 questions), global network (3 questions), performance improvement skills (3 questions), and basic attitude competency (4 questions). The tool was modified to evaluate competency improvement for each question, which was assessed with a 5-point Likert scale ranging from 0 (not improved) to 4 (improved); the higher the score, the higher global leadership competency improvement compared to that before participation in the program. When this tool was developed, factor analysis was conducted to verify its validity; the construct validity was verified by explaining 63% of the total items; in subthemes, Cronbach α=.68-.78 [16]. In this study (all items), Cronbach α=.98.

#### Quality of Program

We used the Student Evaluation of Educational Quality [18] to evaluate university lectures, which consists of 35 evaluation items in 9 themes (Cronbach α=.88-.97): (1) learning and value of lectures, (2) enthusiasm of instructors, (3) structures of lectures, (4) interaction between groups, (5) personal relationship formation, (6) scope of learning content, (7) tests, (8) assignments, and (9) levels of burden and difficulty. We selected 10 items to evaluate the value of learning, instructors, and group activities; each item was assessed with a 5-point Likert scale ranging from 1 (not at all) to 5 (very much). A higher point indicated a better evaluation of the educational program. In this study (all items), Cronbach α=.89.

#### Development of the Virtual International Collaborative Learning Program

**Figure 1:**
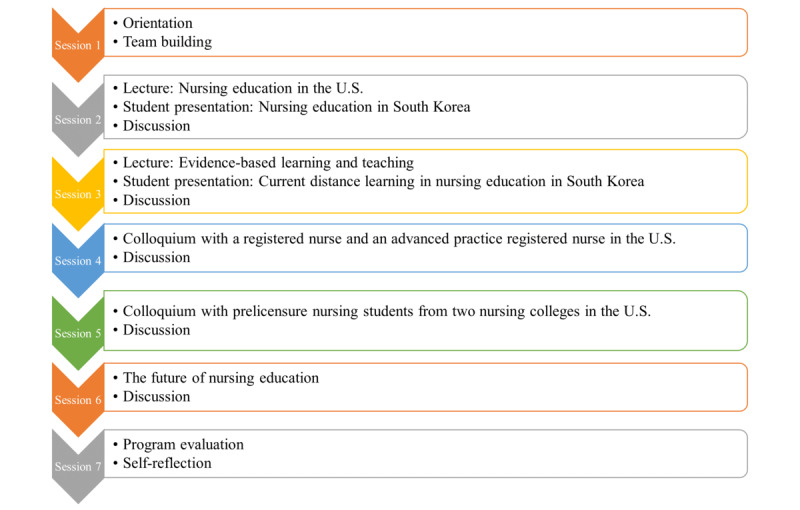
Online international collaborative learning program.

The Office of International Affairs at Ewha Womans University funds an international exchange program each semester; a lead professor plans, supervises, and facilitates the entire program, and students receive a small scholarship upon completion of the program. However, due to the spread of COVID-19, the program was conducted using a videoconferencing platform (Zoom, Zoom Inc). The program was operated by the lead professor in collaboration with a nursing professor at Duke University in the United States; the program consisted of 7 synchronous sessions (running 60 to 90 minutes per session) presented from November 17 to December 22, 2020 (Figure 1). The first session provided an overall program orientation—introduced the program, formed groups, and selected group activity topics. In consideration of the students' varying levels of clinical practice experience, they were placed into 1 of 5 groups (of 3 to 5 students); each group prepared presentation materials 5 times through group activities. Students were expected to submit individual activity reports that consisted of a summary of the day’s program, details of activities, and questions to evaluate the program. In the second and third sessions, students attended special lectures by a professor in the United States on the current status of and latest trends in US nursing education. The students gave presentations on the South Korean nursing education system, analyzing and comparing nursing education in the United States with that in South Korea. In the fourth session, students had a colloquium with a registered nurse and an advanced practice registered nurse in the United States about the role of nurses and nurse specialists and their working experiences in the United States, with a question-and-answer session, and discussed the role of nurses in different medical environments. In the fifth session, the characteristics of clinical practice education in the United States and South Korea were compared and critically analyzed by exchanging stories with undergraduate students in the nursing colleges in the United States. In the sixth session, students had a forum with a professor and discussed the postpandemic future of nursing education as well as their personal plans and goals. The seventh session consisted of a meeting to evaluate the program.

### Data Collection

Data were collected from December 23 to 29, 2020 from college-level nursing students who had completed the program. Quantitative data were collected through a web-based survey using Google Forms, and respondents were asked to complete the survey after providing consent to participate in the study. Focus group interviews were conducted via Zoom by a researcher who was not involved in supervising the educational program. Although the number of participants in a focus group interview varies depending on the literature, it has been found that 7 to 10 participants per group are desirable [19]; thus, 2 groups of 7 or 8 participants (for a total of 15 participants) were formed for 1-hour interview sessions. The interview constituted the relationship-building stage and began with participants introducing themselves to one another and having a casual conversation to break the ice; next students were asked to respond to a wide range of questions or requests, such as “Tell me how you felt while participating in the program?” Participants were encouraged to speak freely about their experiences and to listen to others’ stories and were prompted to answer further questions: “How have you changed after the participation in this program? What kinds of difficulties did you experience during the program? How did this program affect your major capability? What types and methods of education do you think would be more effective? Can you tell us how this program can be more improved?”

During the interview, the interviewer summarized answers to the questions, asking whether the summary was accurate and if anything had been missed. Data collection through interviews continued until the meaning of experiences and subject matter reached theoretical saturation [20]. After each focus group interview, the researcher who had conducted the interview watched and listened to the interview session several times, in order to transcribe the contents as exactly as possible, and confirmed the meaning by reading the transcribed text. The finished transcription was reviewed by a lead professor to ensure accuracy.

### Data Analysis

#### Quantitative Analysis

Quantitative data were analyzed using SPSS statistical software (version 23.0; IBM Corp). The general characteristics of participants were represented by frequency and percentage or mean and standard deviation. Global leadership competency improvement (on a 72 point-scale) and program quality were represented by mean and standard deviation. Global leadership competency improvement and participant characteristics were analyzed using a nonparametric method (Mann-Whitney *U* test).

#### Qualitative Analysis

The focus group interviews were analyzed using a qualitative content analysis method [21]. Researchers repeatedly read the transcribed data to understand the meaning of participants’ thoughts and reflections about the effectiveness of the program. Each researcher read the data and extracted meaningful phrases and sentences containing key concepts. They read the extracted main text, recorded abbreviated semantic units to create proper titles, grouped similar words and compared their differences, and then extracted more abstract categories after discussion with the other researchers. After returning to the original data, they analyzed the transcripts as a whole to confirm the credibility of the category. The research results were shown to all study participants, who confirmed that their experiences in the program were well reflected.

### Rigor of Research

We attempted to ensure the quality of research in terms of credibility, applicability, consistency, and neutrality, which are suggested criteria for evaluating the rigor of research [22]. As co-researchers reviewed and analyzed the data collaboratively, they evaluated and discussed whether the participants’ statements had been converted into appropriate academic terms. In pursuit of credibility, during the integration and analysis of the data, they returned to the participants’ original statements, reviewing whether the analysis results and the interview contents were consistent. For the confirmation of applicability, they shared the research results with a nursing professor with experience in qualitative research who had not participated in this study. During the process, the co-researchers fully understood the data analysis methods and maintained consistency. To ensure neutrality, a researcher who was not in charge of this program conducted interviews with participants, and the co-researchers continuously confirmed and discussed the interview narratives to prevent researchers’ experiences and emotions from influencing the analysis.

### Ethical Considerations

This study was conducted after obtaining approval from the institutional review board of Ewha Womans University (202012-0009-01). Participants were allowed to voluntarily access the web-based survey link. At the beginning of the survey, we provided instructions on the purpose of the study, contents, procedures, audiorecording for the interviews, anonymity of the data, and the right to withdraw participation at any time; after that, informed consent was obtained when the user clicked to indicated whether or not they agreed to participate in the study. Confidentiality and anonymity were maintained during data collection and analysis; personal information was not revealed when recorded data were transcribed. Collected data will be discarded after being stored for 3 years.

## Results

### Participant Characteristics

Of the 15 students who agreed to participate, the data from 14 participants were used for quantitative data analysis; data were omitted from 1 participant due to a missing response on the questionnaire. All participants were female (Table 1).

**Table 1:** Participant characteristics.

Characteristic				Value (n=14)
Age (years), mean (SD)				22.5 (1.16)
**Gender, n (%)**				
	Male			0 (0)
	Female			14 (100)
**Level, n (%)**				
	Sophomore (2nd year)			3 (21)
	Senior (4th year)			11 (79)
**Reasons for program participation, n (%)^a^**				
	Acquire new knowledge			6 (21)
	Personal achievement			11 (39)
	Cultural contact			6 (21)
	Intellectual curiosity			5 (18)
**Previous study abroad program participation, n (%)**				
	No			6 (43)
	Yes			8 (57)
	**If *Yes*, length of study abroad program experience (days), n**			
		≤5	3	
		6-9	1	
		≥10	4	

^a^Multiple answers were possible.

### Global Leadership Competencies

Mean improvement of global leadership competencies was 51.1 (SD 17.9) (Table 2). The most improved subtheme was *open attitude toward diversity*, which increased by a mean of 12.7 (SD 3.9 points) (on a possible range of 0 to 16 points) compared to the preprogram period. Analysis of the relationship between the characteristics of the participants and improvement in global leadership competencies showed no significant difference (*P*=.39) for second- or fourth-year students. There was a statistically significant difference in the subtheme *performance improvement skills* in improvement in global leadership competencies, depending on whether or not the participants participated in face-to-face international exchange programs before (*z*=2.08, *P*=.04).

**Table 2:** Improvement of global leadership competencies.

Theme and subtheme			Score, mean (SD)		Undergraduate level									Experience in international exchange programs						
			All (n=14)		Sophomore (n=3)		Senior (n=11)		*z* score		*P* value		Yes (n=8)			No (n=6)		*z* score		*P* value
**Global leadership competencies**			51.1 (17.9)		45.0 (17.1)		52.7 (15.6)		0.86		.39		57.9 (15.1)			42.0 (18.4)		1.81		.07
	Global mind	8.5 (2.4)		8.0 (1.7)		8.6 (2.6)		0.45		.63		9.4 (2.1)			7.3 (2.4)		1.45		.15	
	Open mind to diversity	12.7 (3.9)		12.3 (3.5)		12.8 (4.2)		0.47		.64		14.0 (2.6)			11.0 (5.0)		1.44		.15	
	Global network	10.4 (4.7)		9.0 (5.3)		12.8 (4.2)		0.71		.48		11.6 (4.6)			8.7 (4.6)		1.31		.19	
	Performance improvement skills	7.9 (3.6)		6.0 (2.7)		8.4 (3.8)		1.18		.24		9.6 (3.0)			5.5 (3.1)		2.08		.04	
	Basic behavioral competency	11.6 (4.1)		9.7 (4.2)		12.2 (4.1)		1.02		.31		13.3 (3.7)			9.5 (4.0 )		1.82		.07	

### Quality of the International Collaborative Learning Program

The mean program quality score was 48.1 points (SD 3.1) (Table 3); among the subitems, clear explanations by faculty, consistency with the program purpose, and instructor friendliness to students showed the highest record with an average of 4.9 points (SD 0.3).

**Table 3:** Student evaluation of program quality.

Program quality items	Mean (SD)
1. Program was intellectually challenging and stimulating.	4.6 (0.6)
2. Learned something considered to be valuable.	4.7 (0.5)
3. The instructor was dynamic and energetic in conducting the program.	4.9 (0.4)
4. The instructor’s explanations were clear.	4.9 (0.3)
5. The learning objectives were in line with the course content.	4.9 (0.3)
6. Students were encouraged to participate in discussions.	4.7 (0.6)
7. Students were encouraged to ask questions and were given meaningful answers.	4.8 (0.4)
8. Students were invited to share ideas and knowledge.	4.8 (0.4)
9. The instructor was friendly towards all students as individuals.	4.9 (0.3)
10. Feedback on group presentation was valuable.	4.9 (0.4)

### Content Analysis of Focus Group Interviews

Content analysis revealed 14 subcategories in the categories *realistic applicability*, *clarification*, *expansion of perspectives*, and *initiative* (Table 4).

**Table 4:** Content analysis of students’ experience in the program.

Categories and subcategories			Example statements
**Realistic applicability**			
	Obtaining answers to realistic questions	“From the standpoint of students, I could learn directly about how the practice was being conducted there [in the US], and I think it was a very good opportunity for comparative studies because I could listen to firsthand experiences of the treatment of nurses there.” “I think it was nice to be able to ask questions about realistic concern[s] such as salary and what it is like living there as a nurse.”	
	Listening to vivid experiences of clinical and practice sites	“I think it was good to be able to hear stories from the standpoint of nurses who are currently working or are students there [in the US].” “I think I got a lot of information because I was able to hear directly what my seniors said, and it was great to feel motivated and comfortable.” “The contents that the professor explained felt more vivid coming from the perspective of the students.”	
	Bridging the difference between direct and indirect experiences	“I have heard a lot of stories about the nursing environment in the US on the internet. Even though I have heard stories from across the globe, it felt very new hearing stories directly from the people in question.” “I thought that there would be many things that would be a little different from the practice in Germany and that it would be hard to absorb the practice fully due to language barriers. But this time was comparatively smoother as it was English-speaking.”	
**Clarification**			
	Demystifying vague aspects of practical problems	“I think the vague fear of the real problem I was concerned about has disappeared to an extent.” “Rather than vaguely thinking about working in the US or abroad, it was nice to be able to think about the process and the real problems that come from it.”	
	Clarifying vague aspects of careers	“In the past, I was only vaguely thinking about working abroad and obtaining very general information about the job of a nurse abroad. However, through this program, I was able to learn, in great detail, what the basis of going abroad should be and what is really needed for it, which has positively changed my attitude toward going abroad.” “I thought it was very helpful because I was motivated a lot, and it seemed like I was stepping closer to something I had just thought vaguely about.” “I was able to solidify my goals a little more, and although they are not perfect, I was able to focus on my future plans.”	
	Solving queries through Q&A	“It was nice to have a lot of time to ask questions to satisfy my usual curiosity. I think it was a good opportunity to learn a lot.” “Being a nurse in the US felt very obscure, but this has helped me feel more hopeful.”	
	Addressing various topics as well as the work aspects of being a nurse	“I believe that learning about real-life issues regarding racism and how to deal with it, in addition to the actual job as a nurse, is a part of education, and this is helpful in that sense.”	
**Expansion of perspectives**			
	Approaching the problem from a self-centered to system-centered perspective	“I also practiced at a university hospital for two years, and I never thought that it was natural for me to learn because this is a teaching hospital. I did not think that the nurses, the patients, or even the department head thought that way, but I was jealous that the American students were able to practice with that mindset. However, knowing that this is a systemic issue, I thought it would be good if it could be improved in Korea as well.”	
	Broadening of international perspectives	“It was very nice to hear about the American system because I was always interested in it but never had the chance to hear about it around me.” “It was nice that the professors and students from the US and Korea each gave presentations; so I could think about the commonalities and differences. It was also nice to broaden my perspective by meeting the other seniors who are in clinics.”	
	Widening of career vision	“Originally, I was thinking of working in a clinic or preparing to work in public service in Korea, but this has helped me expand my vision and also consider being a nurse in the US.” “I also liked being able to hear more practical stories, and frankly, I had little thought of being a nurse in the US, but listening to these stories made me feel more interested and like I have a wider choice of options.” “I had dismissed my thoughts for a while because I was worried about getting a job at a Korean hospital after graduating, but it was nice to have a broader vision because of this opportunity.”	
	Expanding relationships through collaborative learning	“It was nice to be able to interact with people who work in the clinic or with students attending school there.” “It seemed like a very good program overall as I was able to interact with many teachers.” “First of all, I think it was a very meaningful time because I could ask questions about nursing education and the system in the US while interacting with professors, nurse, advanced practitioner registered nurse, and other nursing students from the US.” “I think it was a meaningful opportunity to be able to communicate with people who are working in the US from whom there is a physical distance otherwise and be able to have my questions answered.”	
**Initiative**			
	Positive changes in job perception and perspective	“I was able to be more open-minded. I was a little skeptical about the attitude of the people around me in the job as a nurse, but learning that this may be because of cultural and environmental differences has made me more hopeful.”	
	Realizing the need for holistic nursing	“The most meaningful part for me was that it made me think about the nature of nursing. I think it was nice to be able to think about holistic nursing once more while listening to experiences of nursing in the US.”	
	Changing to a positive attitude for the development of nursing	“Interacting with the American students has made me think that I should make a more active effort to develop nursing skills. I think I will be more proactive in thinking about and acting on problems in the future.” “I think there are thoughts and cultures exclusive to nurses in Korea; so even if I went to the US to study and experience their culture, I think I would like to come back to Korea to make changes in better directions.” “I also thought that I wanted to change the image and status of Korean nurses. “It was a meaningful time to think about what needs to be developed in Korea while interacting with the nurses and students.”	

## Discussion

### Principal Findings

We sought to evaluate the effectiveness of an online international collaborative program for nursing students by assessing global leadership competencies and program quality. Participants’ global leadership competencies improved compared to before their participation in the program, and improvement in *openness to diversity* was particularly high among the subthemes. The findings are in line with those of other studies on face-to-face programs, for example, international exchange programs for nursing college students in Vietnam and South Korea consisting of lectures and visits to local hospitals and nursing education institutions contributed to developing global leadership competencies, understanding cultural diversity, and keeping an open mind [13]. Similarly, a short-term program abroad to increase global health competencies significantly raised nursing students’ global leadership competencies [9,23] led to increased open-mindedness toward other people and cultures (the most remarkable change in competencies based on analysis of daily records) [9]. The program included presentations on the US nursing system and group presentations on the South Korean nursing system; students had opportunities to discern differences in health care and clinical practice systems through group discussions and question and answer sessions. The findings of our study indicate that such factors enabled them to accept and have more respect for diversity. Furthermore, our study confirmed that collaborative learning conducted online in lieu of field visits helped students improve their global leadership by allowing them to hear experiences described directly by field nurses and other nursing school undergraduate students.

There was no significant difference in improvement in global leadership competencies between students who had participated in face-to-face international exchange programs before and those who had not (*P*=.07). This is consistent with the findings of a previous study [24] in which students with participation experiences showed higher global leadership than those without such experiences, but the difference was not statistically significant. In our study, improvement in *performance improvement skills* in global leadership competencies was significantly higher (*P*=.04) for students with international exchange program participation experiences than that for students without these experiences. In this study, performance improvement skill was described as the capability to set organizational goals and utilize necessary information and resources to achieve results. Students with experiences showed more improvement due to synergistic effects of knowledge and information newly acquired through this program combined with knowledge and experiences acquired through previous international collaborative activities. Given these results, it seems necessary to continue to develop and implement similar international programs that can cultivate global competencies in nursing students; however, because this was a single-group postdesign study, quasi-experimental studies are needed to verify the effects of improving global leadership competencies.

Focus group interview analyses revealed that participants experienced realistic applicability through the program. In the web-based learning environment, realism is an important factor for enhancing learning effects [25]. As the program operated in real time, participants could listen to field stories told directly by field nurses and students, who were able to answer their questions about information previously obtained through lectures. Participants stated that the vivid field stories offered by students studying in US clinical and nursing education fields made their learning a valuable experience. International exchange programs included local visits to reinforce realism for nursing students [9,10,26]; therefore, it was meaningful to investigate whether there was a relationship between the effects of an online international program and realism. In particular, opportunities for students to listen to stories told directly by nurses or nurse specialists working at medical sites overseas about their work and roles can be considered as a means of increasing the sense of reality, which can otherwise be limited in web-based programs.

Participants expressed that their concerns and uncertainties about their future careers were addressed or clarified by listening to field stories about the clinical experiences of US nurses and nurse specialists and the practical experiences of students in the United States. It can be said that this program helped participants to refine the details and objectives of their career paths and specify active plans to achieve them. According to adaptive career decision-making theory, career decisions can be molded by personal experiences and stimuli from the outside world [27]. An international collaborative learning program such as that described in our study can assist fourth-year nursing college students in specifying their career paths by broadening their career horizons and providing specific information about how to realize their goals. Additionally, the program in the study included diverse topics: trends in US nursing education, comparison and analysis of nursing education systems in the United States and South Korea, role expectations in different medical settings based on stories from US nurses or nurse specialists, and comparison and analysis of clinical practice education through collaborative activities with local students. As shown in previous studies, participants experienced an increase in knowledge, thought, international perspective [10], and view of career paths and human relations [9]. The participants expressed that their international perspective was particularly broadened because the learning tasks undertaken prior to direct exchanges had provided a comparison and analysis of the South Korea and US systems. Findings support those of a previous study [28] that explored a prediction model of student achievement, which suggested that the assignment of learning tasks in web-based lectures can be an important factor affecting learning outcomes. The passive web-based learning process in this study was likely complemented by prior tasks and reinforced learners' self-directedness, further maximizing such expandability. The effects of self-confidence and expanded cognition [26] through international exchanges became possible via active question and answer periods and practical answers in the web-based environment.

Participants showed initiative in changing themselves and their environment through this program. After listening to field stories from the United States, they indicated that they recognized the need for comprehensive nursing care practices that respect patients’ emotional and cultural needs. This result supports those from a previous study [29] in which nursing college students in clinical practices in US hospitals found, through web-based training, that patients in hospitals relied almost entirely on nurses who provided comprehensive nursing care. The participants additionally realized that perceived negative attitudes toward the nursing profession could be due to cultural differences rather than the nature of the profession; this realization led them to consider their profession more positively. Furthermore, their attitude toward nursing development became more positive as they perceived the image, status, and efforts of South Korean nurses more positively. This change in attitude is in line with results of a previous study [9] in which participants demonstrated greater pride in the nursing profession after participating in a short-term program abroad to increase their global health competencies. The participants in our study were able to accumulate more nursing knowledge as well as expand their psychodynamic perspective regarding its utilization in the field.

Students evaluated program quality highly overall, but the item on whether the program was intellectually challenging and stimulating had the lowest score. This could be improved by assessing the topics of interest of participating students before developing the program, including activities on identifying current relevant issues, and discussing them. As for the evaluation of the operation process, the strength of the program was that it was conducted online to allow students to participate during the semester without temporal and spatial constraints and burden of cost. Active interactions between learners and the outside world are important for higher learning effects [25]. When interactions are not active, there can be problems such as decreased immersion, lecture dissatisfaction, and an increase in dropout rates [30]; therefore, the program utilized a videoconferencing platform capable of interactive communication that encouraged students to participate in real-time collaborative activities actively, thereby enhancing the sense of immersion and quality of the program. The evaluation showed that discussing and preparing presentation materials in small groups allowed participants to improve their understanding of corresponding topics at each session and that there was a lack of time for 60-minute sessions with local nursing students. There was some confusion due to sessions progressing on different days of the week; therefore, we suggest that the next program should devote extended time to the program and utilize fixed days of the week for sessions. Evaluation of program content included requests for seminars with field nurses who had more diverse backgrounds or experiences (eg, work in different wards or attended graduate school), and for program levels subdivided by year in college (eg, freshman through senior).

This study has several strengths. The effectiveness of an online international collaborative learning program for nursing college students, developed within the restrictions imposed by the COVID-19 pandemic, was verified by combining quantitative data (surveys) with qualitative data (focus group interviews).

### Limitations

Limitations exist because this was a single-group postdesign study, and there is no feasibility study for pre and postcomparison verification. It is necessary to conduct research to confirm the effectiveness of the program based on practical experiences and college grade levels of student participants. In addition, because most research on international exchange programs has focused on field trips, there were limited tools to verify the effectiveness of our web-based programs; therefore, it is necessary to develop a tool that reflects the characteristics of distance education to measure the effectiveness of the program.

### Conclusions

Previous research on international exchange programs has focused on field trips, yet this study examined the program effectiveness of an online international collaborative learning program for nursing college students. We confirmed the effectiveness of the program in improving global leadership competencies during the COVID-19 pandemic, which had restricted the ability to operate traditional exchange programs between countries. We suggest conducting follow-up studies to verify the mid- to long-term intervention effects of continuous operation of the program, rather than one-off training, after planned incorporation into the nursing education global nursing course curriculum. We further suggest developing programs in connection with various organizations that utilize the advantages of web-based learning environments.

## References

[1] Kim T The effects of covid-19 and countermeasures for social policy. Kihasa.

[2] Al-Balas M, Al-Balas HI, Jaber HM, Obeidat K, Al-Balas H, Aborajooh EA, Al-Taher R, Al-Balas B (2020). Distance learning in clinical medical education amid COVID-19 pandemic in Jordan: current situation, challenges, and perspectives. BMC Med Educ.

[3] Bozkurt A, Sharma R (2020). Emergency remote teaching in a time of global crisis due to CoronaVirus pandemic. Asian J Distance Educ.

[4] Martinez J (2020). Take this pandemic moment to improve education. EdSource.

[5] Mishra L, Gupta T, Shree A (2020). Online teaching-learning in higher education during lockdown period of COVID-19 pandemic. Int J Educ Res Open.

[6] Sun L, Tang Y, Zuo W (2020). Coronavirus pushes education online. Nat Mater.

[7] Baek D (2020). Online education innovation 'break through' the postcorona era. DH News.

[8] Kang S, Nguyen TAP, Xippolitos L (2015). Analyzing educational needs to develop an undergraduate global health nursing program. J Nurs Educ Pract.

[9] Hwang SY, Kim JS, Ahn H, Kang SJ (2015). Development and effect of a global health capacity building program for nursing students. J Korean Acad Commun Health Nurs.

[10] Yun ES (2018). Korean nursing students experiences of a short-term health and medical overseas study abroad program. Korean Assoc Learner-Centered Curric Instr.

[11] (2008). Cultural competency in baccalaureate nursing education. American Association of Colleges of Nursing.

[12] (2020). Nursing education certification evaluation standard book. Korean Accreditation Board of Nursing Education.

[13] Kang S, Trang Ho TT, Phuong Nguyen TA (2016). Effects of a global health nursing program on Vietnamese and South Korean students. J Nurs Educ Prac.

[14] Lee H, Kim HS, Cho E, Kim S, Kim J (2015). Global health competencies for undergraduate nursing students in Korea. J Korean Acad Soc Nurs Educ.

[15] Frisch NC (1990). An international nursing student exchange program: an educational experience that enhanced student cognitive development. J Nurs Educ.

[16] Nam KY (2011). Importance-performance analysis on global leadership competency-focus on employees in global enterprises and college students. Hanyang University.

[17] Song YS (2010). Global leadership competencies for human resource development in Korean enterprises.

[18] Marsh HW (1982). SEEQ:a reliable, valid, and useful instrument for collecting students? evaluations of university teaching. J Educ Psychol Feb.

[19] Kim S, Kim H, Lee S, Lee K (2000). Focus Group Methodology 2nd edition.

[20] Strauss A, Corbin JM (1998). Basics of Qualitative Research.

[21] Elo S, Kyngäs H (2008). The qualitative content analysis process. J Adv Nurs.

[22] Guba EG, Lincoln YS (1994). Competing paradigms in qualitative research. Handbook of Qualitative Research.

[23] Hwang WJ, Jo HH (2020). Development and application of a program for reinforcing global health competencies in university nursing students. Front Public Health.

[24] Chun Y, Kim K, Hwang Y (2017). The effect short-term foreign volunteer activity of global leadership on university students. Asia Pac J Multimed Serv Art Humanit Sociol.

[25] Doo MY, Kwon H, Moon E (2017). A meta-analysis of the effects of presence on learning performance in online learning environment. Korean J Educ Methodol Stud.

[26] Jeong HS (2017). Korean nursing students' experiences of a short-term study abroad program in an American college of nursing. J Qual Res.

[27] Krieshok TS, Black MD, McKay RA (2009). Career decision making: the limits of rationality and the abundance of non-conscious processes. J Vocat Behav.

[28] Jho H (2018). Exploration of predictive model for learning outcomes of students in the e-learning environment by using machine learning. Korean Assoc Learner-Centered Curric Instr.

[29] Seo KS, Kim JA (2017). Clinical practicum experience among South Korean nursing students in the US hospitals. J Qual Res.

[30] Boston WE, Ice P (2011). Assessing retention in online learning: an administrative perspective. Online J Dist Learn Admin.

